# Pharmacological Rac1 inhibitors with selective apoptotic activity in human acute leukemic cell lines

**DOI:** 10.18632/oncotarget.21533

**Published:** 2017-10-04

**Authors:** Maia Cabrera, Emiliana Echeverria, Federico Remes Lenicov, Georgina Cardama, Nazareno Gonzalez, Carlos Davio, Natalia Fernández, Pablo Lorenzano Menna

**Affiliations:** ^1^ Instituto de Investigaciones Farmacológicas, Facultad de Farmacia y Bioquímica (ININFA-UBA CONICET), Buenos Aires, Argentina; ^2^ Instituto de Investigaciones Biomédicas en Retrovirus y SIDA, Facultad de Medicina, (INBIRS-UBA-CONICET), Buenos Aires, Argentina; ^3^ Laboratorio de Oncología Molecular, Universidad Nacional de Quilmes, Buenos Aires, Argentina

**Keywords:** Rac1, acute myeloid leukemia, apoptosis, ZINC69391, 1A-116

## Abstract

Rac1 GTPase has long been recognized as a critical regulatory protein in different cellular and molecular processes involved in cancer progression, including acute myeloid leukemia. Here we show the antitumoral activity of ZINC69391 and 1A-116, two chemically-related Rac1 pharmacological inhibitors, on a panel of four leukemic cell lines representing different levels of maturation. Importantly, we show that the main mechanism involved in the antitumoral effect triggered by the Rac1 inhibitors comprises the induction of the mitochondrial or intrinsic apoptotic pathway. Interestingly, Rac1 inhibition selectively induced apoptosis on patient-derived leukemia cells but not on normal mononuclear cells. These results show the potential therapeutic benefits of targeting Rac1 pathway in hematopoietic malignancies.

## INTRODUCTION

Rho-GTPases are molecular switches that cycle between an inactive GDP-bound form and an active GTP-bound form. This cycle is closely regulated by guanine nucleotide exchange factors (GEFs) that catalyze nucleotide exchange and mediate activation [1]; and GTPase-activating proteins (GAPs), that stimulate GTP hydrolysis and inactivate the GTPase [2]. Only the active GTP-bound state binds to downstream effector proteins and actively transduces signals [3]. Rac1 is one of the most studied members of Rho-GTPases family and controls fundamental cellular processes including cell proliferation, actin cytoskeleton reorganization, migration, cell cycle progression, cell adhesion, differentiation and apoptosis [4–6].

Acute myeloid leukemia (AML) is an aggressive blood disorder characterized by an accumulation of immature hematopoietic stem cells in the bone marrow [7, 8]. AML is the most common type of leukemia in adults with lowest survival rate of all leukemias.

Due to their key role in different cancer types, Rho GTPases are attractive and validated targets for anticancer therapies [9–11]. Rac1 GTPase acts as critical mediator of signaling pathways contributing to the interactions of hematopoietic stem cells with their microenvironment [12]. This protein is overexpressed in primary acute leukemia cells and leukemia stem cells compared to normal bone marrow mononuclear cells [13–15]. The siRNA-mediated knockdown of Rac1 expression in leukemia cell lines proved to inhibit cell proliferation, migration and colony formation [15]. In contrast, overexpression of Rac1 increases cell migration and proliferation potential of leukemic cells, which could be implicated in leukemia development and progression [16, 17]. All this evidence supports the therapeutic potential of target-based therapies against Rac1-GTPase in hematological malignancies.

Previously, we identified ZINC69391 and its analog 1A-116 as small molecules that inhibited Rac1-GEF interactions reducing Rac1 activation levels on different cancer cells. ZINC69391 was able to inhibit cell proliferation, cell cycle progression and migration of highly aggressive breast cancer cell lines. Moreover, ZINC69391 and 1A-116 inhibited lung metastasis *in vivo* [18]. We also described an antiapoptotic and anti-invasive effect of ZINC69391 on glioblastoma cell lines [19].

**Figure 1:**
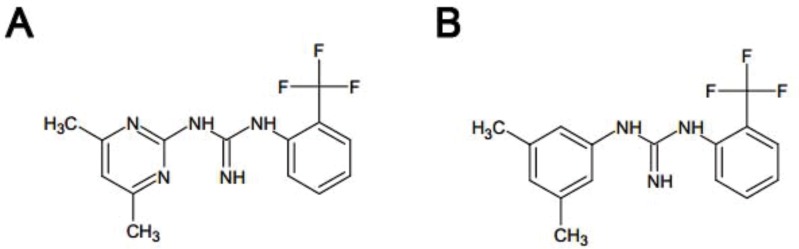
Chemical structures of Rac1 inhibitors **(A)** Chemical structure of ZINC69391 (C14H15F3N5; molecular weight, 310.303). **(B)** Chemical structure of 1A-116 analog (C16H16F3N3; molecular weight, 307.31).

In the present work we studied the antitumoral activity of the pharmacological Rac1 inhibitors, ZINC69391 and 1A-116, (Figure 1) in a panel of human acute leukemia cell lines, representing different levels of maturation. We demonstrate that ZINC69391 treatment induced growth inhibition associated to apoptotic cell death program activation. This pro-apoptotic activity involved a pronounced caspase 3 activation, mitochondrial membrane potential loss, sequential caspase 9 and 8 activation and increase of the phosphorylated fraction of Bcl-2. In line with our previous results, 1A-116 also showed to be a more potent agent on leukemic cells. Interestingly, Rac1 inhibition displayed selective activity on patient-derived leukemic cells having no cytotoxic effect on normal monocytic and lymphocytic cells, representing a promising pharmacological and selective compound for the treatment of hematological malignancies.

## RESULTS

### ZINC69391 is a small molecule drug that inhibits growth of human leukemia cell lines

ZINC69391 is a first generation small molecule that was identified as a Rac1-GEF interaction inhibitor, using a docking-based virtual library screening approach. In previous reports, ZINC69391 was able to inhibit several Rac1-GEF interactions, which were associated to antiproliferative effects, cell cycle arrest and migration inhibition of highly aggressive breast cancer cell lines [18]. Moreover, ZINC69391 demonstrated *in vivo* anti metastatic activity in lung and apoptotic induction in glioma cells with decreased cell migration and invasion [19]. Based on these previous reports we sought to determine whether ZINC69391 exhibited activity against cell proliferation *in vitro* on a panel of human acute leukemia cell lines with different stages of cell differentiation. Three human myeloid leukemia cell lines (U937, HL-60 and KG1A) and a leukemia-derived T-cell line (Jurkat cells) were treated with ZINC69391 for 48h. Cell growth was inhibited in a concentration-dependent manner showing IC50 values of micromolar range (Table 1). This result indicates a high potency of ZINC69391 as a proliferation inhibitor of leukemic cells. Interestingly, Jurkat cells exhibited a shift in the concentration-response curve in comparison to myeloid lineage, suggesting a lower sensitivity of Jurkat cells to ZINC69391 although the maximal response was similar to that observed for KG1A.

**Table 1:** IC_50_ values of ZINC69391 in human acute leukemia cell lines

Cell line	U937	HL-60	KG1A	Jurkat
**IC_50_μM (CI_95_)**	43,4 (38-49)	41,7 (37,4-46,6)	54,1 (41-71)	41 (29,6-58,5)

### ZINC69391 treatment increases G2/M subpopulation in AML cell lines

**Figure 2:**
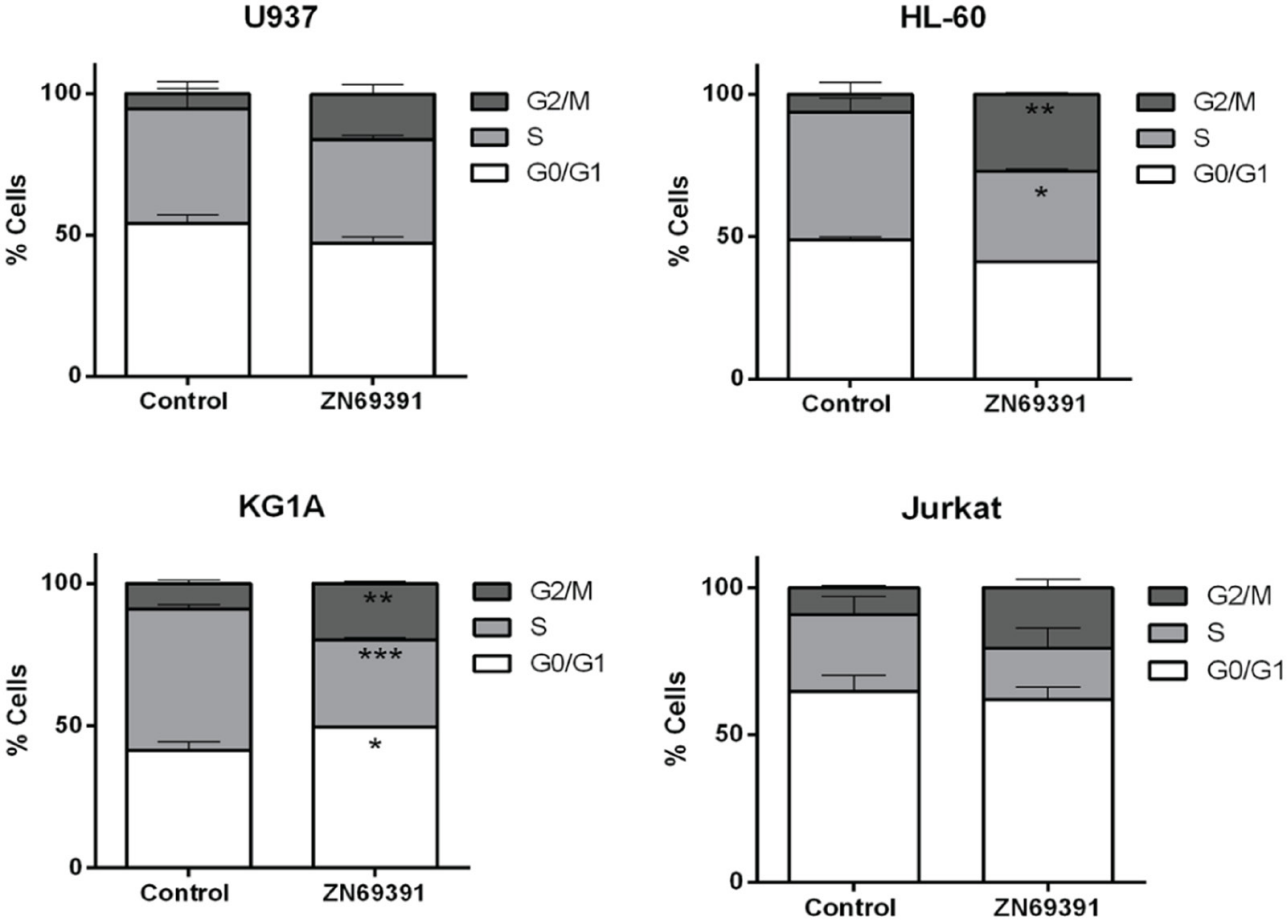
Cell cycle distribution after ZINC69391 treatment in human acute leukemia cell lines Synchronized G0/G1 cells were exposed to ZINC69391 50 μM or to 0.05% (v/v) DMSO, vehicle control group for 24h. Cell cycle distribution was calculated as described in Material and Methods. Data represent the mean ± SD (n > 3). ^*^p< 0.05.

Regarding the involvement of Rac1 on cell cycle progression we next compared the effects of ZINC69391 on cell cycle distribution. Synchronized HL-60, U937, KG1A and Jurkat cells were treated for 24h with 50 μM ZINC69391 and then stained using PI to establish DNA content by flow cytometry. The antiproliferative effects of ZINC69391 correlated with effects over cell cycle progression showing a significant increase in G2/M subpopulation for HL-60 and KG1A cell lines (Figure 2). Although for U937 and Jurkat cell lines the differences were not significant, the same tendency was observed. These results are in concordance with several reports describing that Rac1 inhibition is associated to a G2/M cell cycle arrest [20–22].

### ZINC69391 triggers apoptosis on human acute leukemic cells

**Figure 3:**
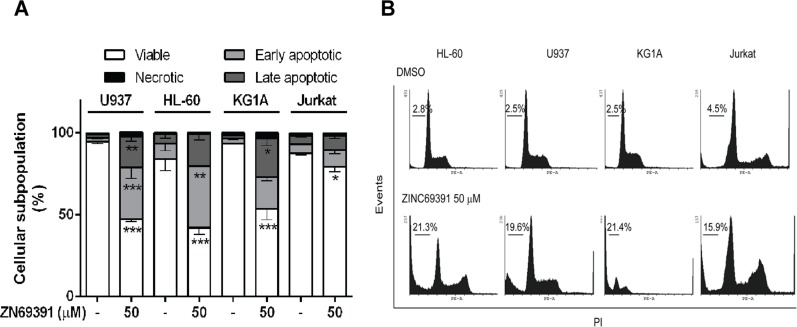
Pro-apoptotic activity of ZINC69391 in human acute leukemia cell lines **(A)** Starved cells were exposed to ZINC69391 50 μM or to 0.05% (v/v) DMSO, vehicle control group. After 24h of treatment, cells were analyzed by flow cytometry to detect exposed phosphatidylserine by annexin V binding. The graphic shows the different cell subpopulations according to the annexin V/PI staining pattern: cells labeled with only annexin V (early apoptosis), cells labeled with annexin V and PI (late apoptosis), and cells labeled only with PI (necrotic cells). Data is presented as mean ± SD from four independent experiments. ^*^p<0.05; ^**^p<0.01. **(B)** Representative histogram of leukemic cell lines where the subpopulation hypodiploid subG0/G1 is observed.

To further investigate the molecular mechanisms underlying antiproliferative effects of ZINC69391 on leukemia cells, different apoptotic markers were evaluated. First, we analyze the change in annexin V/PI positive staining of cells by flow cytometry. Using the same cell panel described before, we found that treatment with 50 μM ZINC69391 for 24h led to a significant increase in apoptotic cells (Annexin V positive) in HL-60, U937 and KG1A cell lines (Figure 3A), while no significant change was observed for Jurkat cells. Interestingly, these results correlate with an increased sub G0/G1 population (apoptotic cells) observed in cell cycle analysis for all cell lines tested (Figure 3B). It is worth noting that ZINC69391 did not promote necrosis at the time tested in any of the cell lines.

**Figure 4:**
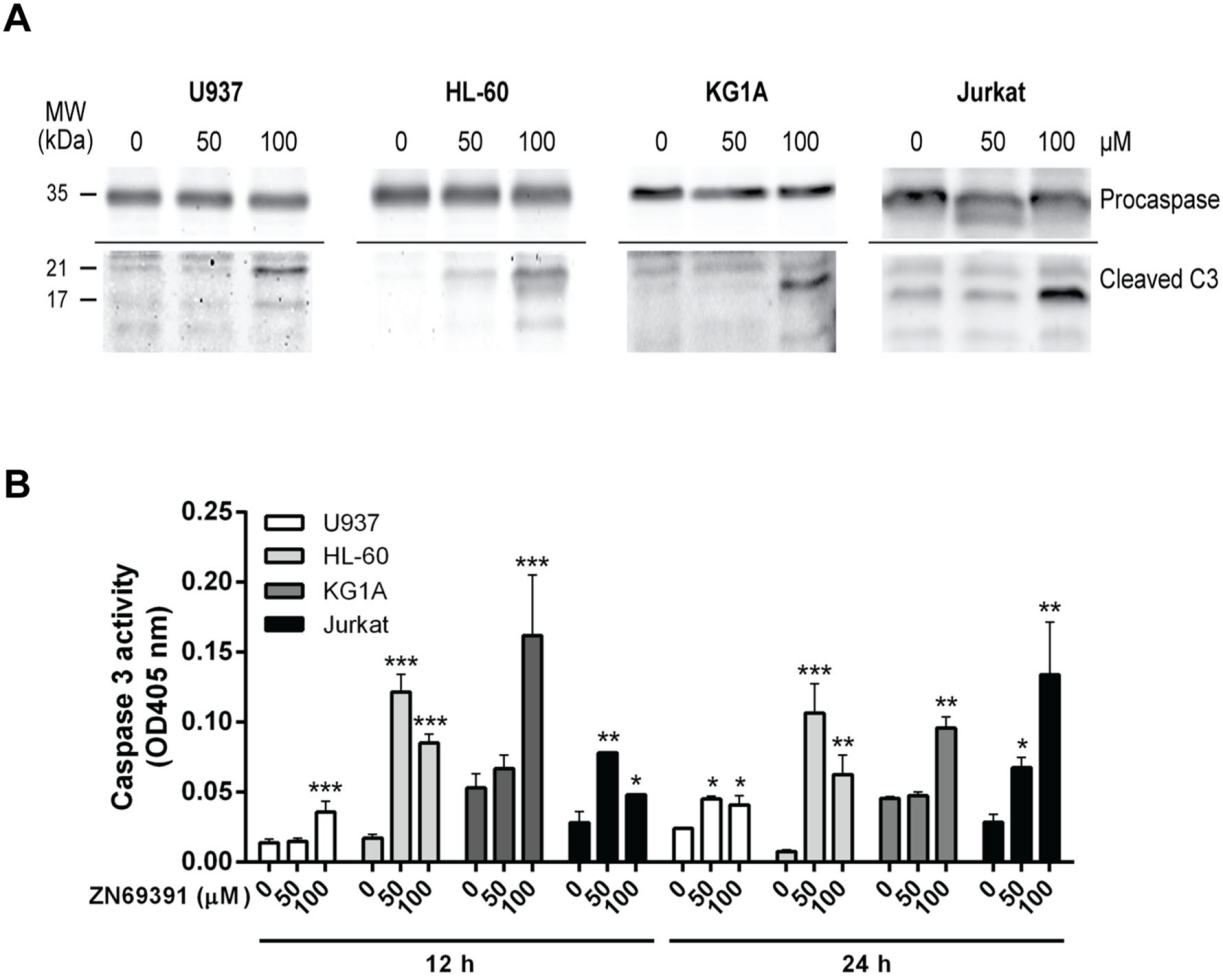
Caspase 3 activation in apoptosis induced by ZINC69391 **(A)** The cleavage of caspase 3 was evaluated by western blot. Equal amounts of protein were subjected to SDS–PAGE and western blot with anti-caspase 3. Data are representative of at least three independent experiments. **(B)** Cells were treated with ZINC69391 in the indicated concentrations and time, and caspase 3 protease activity was measured using a colorimetric kit as described in Materials and Methods and expressed as OD405nm values. Data are presented as mean ± SD from four independent experiments. ^*^p<0.05; ^**^p<0.01; ^***^p<0.005.

Since caspase 3 is a terminal effector in the apoptotic cascade that is activated after proteolytic cleavage, we assessed cleaved caspase 3 by western blot after ZINC69391 treatment. After 24h incubation with ZINC69391, cells exhibited an increase in both active fractions of caspase 3 (Figure 4A). Next, we confirmed caspase 3 activation by a colorimetric assay using ZINC69391 50 μM and 100 μM for different incubation periods (Figure 4B). Incubation with ZINC69391 augmented the enzymatic activity of caspase 3 in a concentration dependent manner, in agreement with the results obtained by western blot (Figure 4A). These results showed that ZINC69391 triggered apoptosis in human acute leukemic cells, as confirmed by three different approaches.

### Rac1 inhibitor ZINC69391 modifies NF-κB transcriptional activity

**Figure 5:**
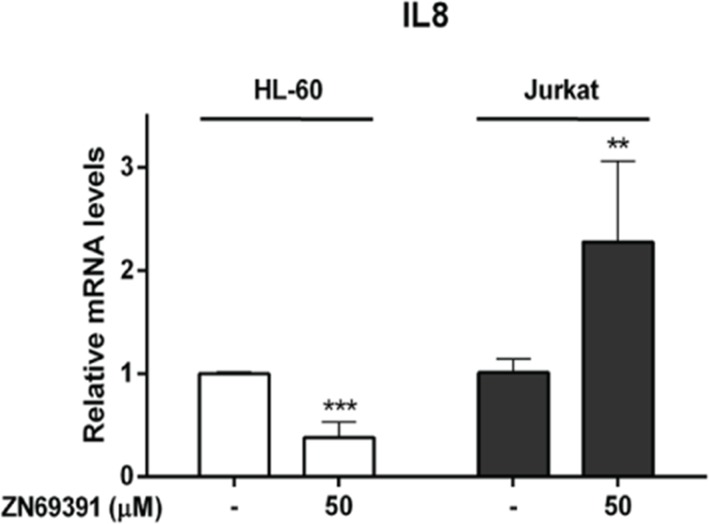
Real time PCR evaluation of Interleukin 8 mRNA levels in leukemic cells treated with ZINC69391 HL-60 and Jurkat cells were incubated with 50 μM of ZINC69391 or 0.05% (v/v) DMSO vehicle control group for 12h. IL-8 mRNA levels were quantified by qPCR as described in the methods section. Results are mean ± SD of at least three independent experiments performed in triplicates. ^**^p < 0.01; ^***^p<0.005.

To further evaluate the implications of Rac1 inhibition, we studied the effect of ZINC69391 on NF-κB transcriptional activity. NF-kappaB is a transcription factor that plays a crucial role in cell cycle progression and expression of anti-apoptotic genes, having Rac1 a major role in its activation [23, 24]. We selected Interleukin 8 (IL-8), a gene transactivated by NF-κB, as a downstream target to be evaluated by qPCR in HL-60 and Jurkat cell lines [25, 26]. We found that ZINC69391 treatment potentiated twice IL-8 mRNA levels in Jurkat cells, while in HL-60, mRNA levels were profoundly diminished (Figure 5A). This opposed response to NF-κB-induced transcriptional activity after ZINC69391 treatment reflects the differential sensitivity observed for these cells lines in previous assays.

### ZINC69391 pro-apoptotic activity involves alterations of mitochondrial effectors and its integrity

**Figure 6:**
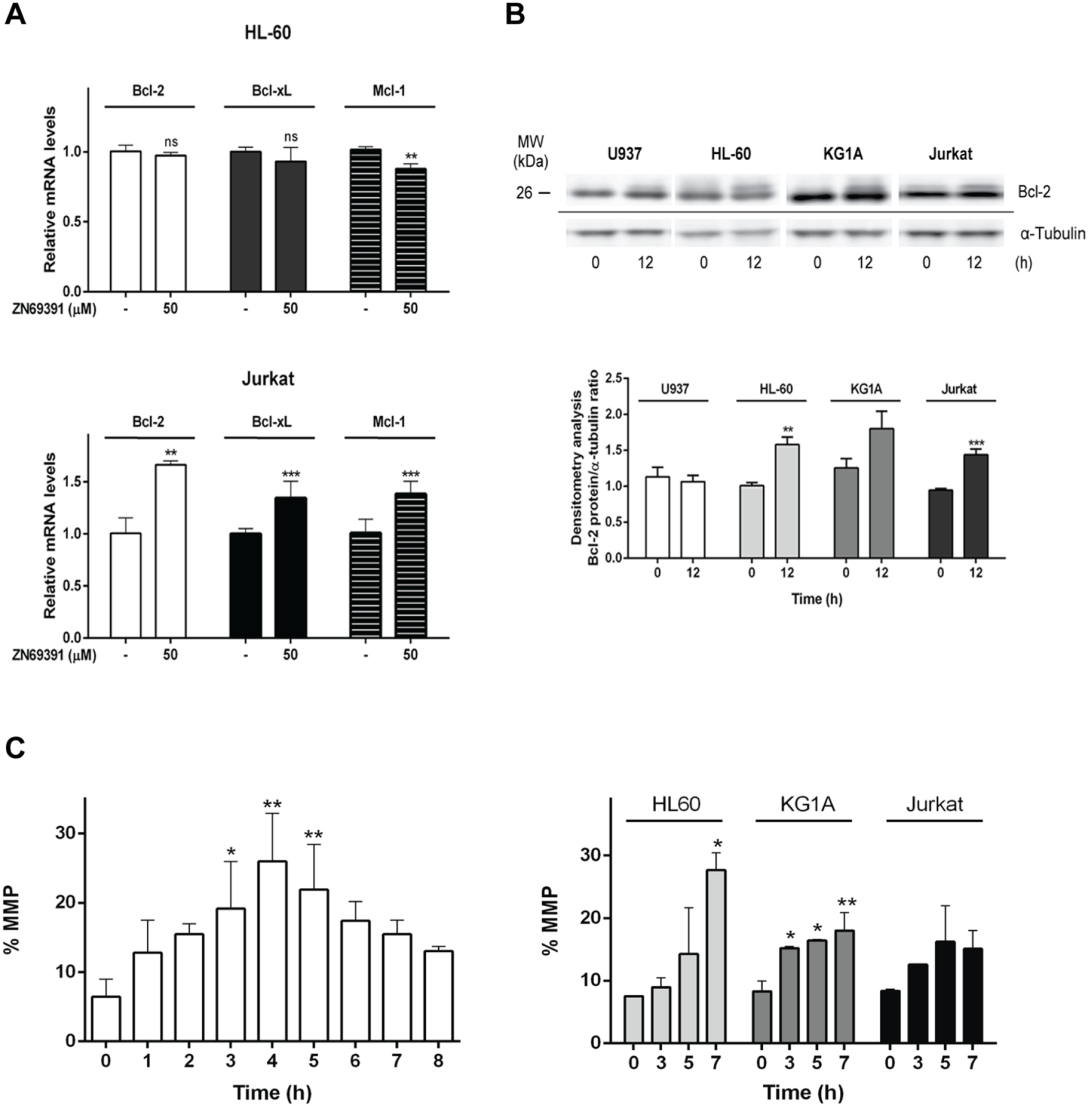
ZINC69391 effect on anti-apoptotic Bcl-2 family proteins and mitochondrial membrane permeability (MMP) **(A)** HL-60 and Jurkat cells were incubated with 50 μM of ZINC69391 or 0.05% (v/v) DMSO vehicle control group for 12h. Bcl-2, Bcl-xL and Mcl-1 mRNA levels were quantified by qPCR as described in the methods section. Results are mean ± SD of at least three independent experiments performed in triplicates. ^**^p<0.01; ^***^p<0.005 **(B)** Analysis of MMP alteration at indicated times for U937 (left panel) and HL-60, KG1a and Jurkat (right panel) cell lines. Cells treated with 50 μM of ZINC69391 or 0.05% (v/v) DMSO vehicle control group, were evaluated for changes in fluorescence intensity of DiOC6 probe by flow cytometry. The bar graph shows differences among treatment times (n=4) ^*^p<0.05; ^**^p<0.01; ^***^p<0.005. **(C)**. Representative graphic shows Bcl-2 protein assessment by Western blot. Equal amounts of protein were subjected to SDS–PAGE and western blot using anti-Bcl-2 protein antibody. ^*^p<0.05; ^**^p<0.01; ^***^p<0.005.

The proteins of Bcl-2 family are regulators of several mechanisms associated to cell death such as mitochondrial permeabilization, apoptosis amplification [27] and more recently, the modulation of cellular redox metabolism [28]. Regarding the fact that Bcl-2 family members function downstream of Rac1 [29] and considering their participation on mitochondrial membrane integrity, we also investigated the effect of ZINC69391 on Bcl-2, Bcl-xL and Mcl-1 mRNA levels by qPCR. After 12h of ZINC69391 treatment, HL-60 cells showed a significant decrease only in Mcl-1 levels, while Jurkat cells exhibited increased mRNA levels of all proteins evaluated (Figure 6A).

Since Bcl-2 family proteins function is also regulated by post translational modifications, we decided to evaluate the protein Bcl-2 by western blot. Immunoblotting showed a shift on Bcl-2 mobility for HL-60, KG1A and Jurkat cells after 12h of ZINC69391 treatment compared to control cells (Figure 6B). Shifts like that were previously associated to Bcl-2 phosphorylated fractions which are a common mechanism of modulation of Bcl-2 activity [30, 31].

**Figure 7:**
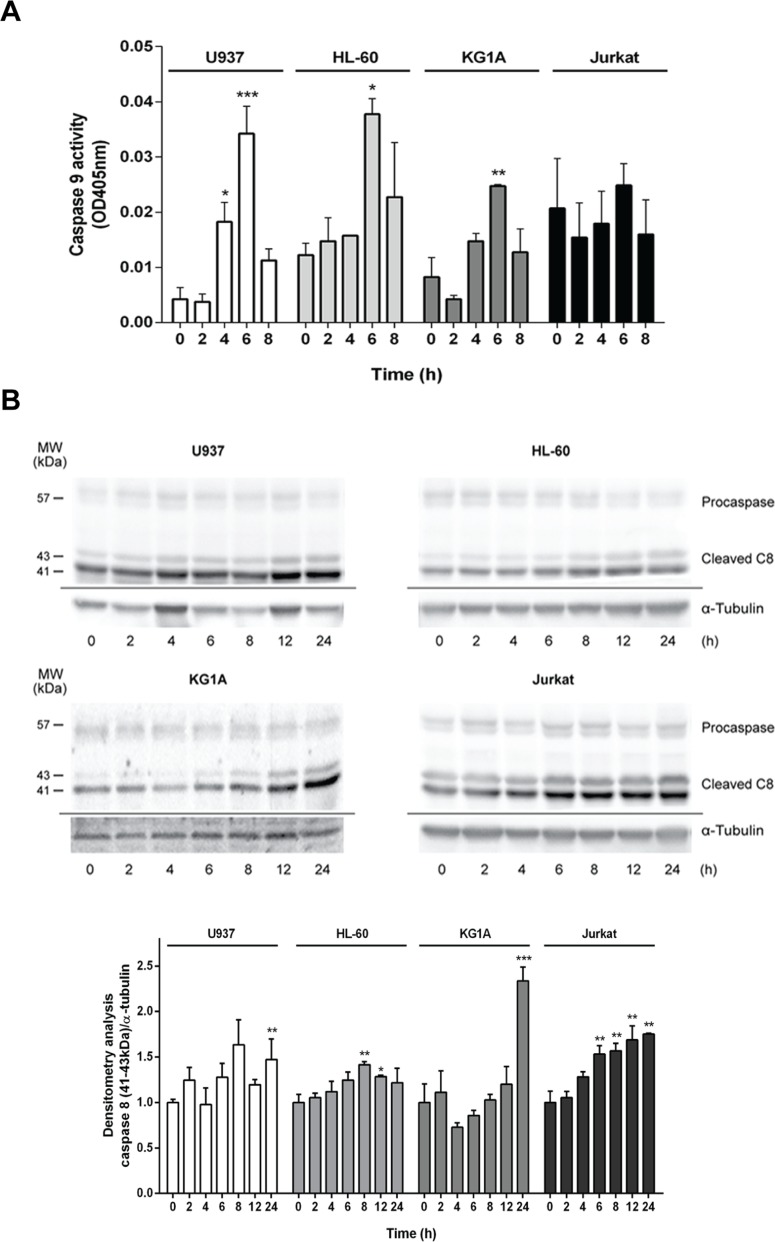
ZINC69391 stimulated activation of caspases 8 and 9 **(A)** Cells were treated with ZINC69391 for the indicated time, and caspase 9 protease activity was measured using a colorimetric kit as described in Materials and Methods and expressed as OD405nm values. Data are presented as mean ± SD from three independent experiments; ^*^p<0.05; ^**^p<0.01; ^***^p<0.005. **(B)**. Cleavage of caspase 8 was evaluated by Western blot. Equal amounts of protein were subjected to SDS–PAGE and western blot using anti-caspase 8 antibody. Blots were subjected to densitometry analysis using ImageJ software. Data are presented as mean ± SD respect to control of at least three independent experiments. ^*^p<0.05; ^**^p<0.01; ^***^p<0.005.

In the mitochondrial or intrinsic pathway of apoptosis, caspase activation is dependent of outer mitochondrial membrane permeabilization induced by pro-apoptotic members of the Bcl-2 family. In order to gain further insight into the mechanism of apoptosis exerted by ZINC69391, we evaluated the effect of the compound on mitochondrial membrane permeability (MMP). Dyes such as 3,3′-dihexyloxa-dicarbocyanine (DiOC6) are lipophilic cationic probes that are taken up by living cells and accumulate in intact mitochondria. When mitochondrial membranes are damaged or leaky there is a loss of DiOC6 fluorescence intensity. Based on that, it can serve as marker of mitochondrial membrane integrity [32]. We treated the cell lines with ZINC69391 for different time periods and then measured DiOC6 fluorescence by FACS. As seen in Figure 6C (left), U937 cells treated with ZINC69391 show an increase in MMP in a time dependent manner, having its maximum effect at 4h post-treatment. Interestingly, this same effect was observed in HL-60 and KG1A cells, while Jurkat showed a modest and not significant increase in MMP process (Figure 6C right). We next evaluated the effect of ZINC69391 on pro-caspase 9 activation, a relevant initiator caspase associated to the mitochondrial or intrinsic pathway. As shown in Figure 7A, ZINC69391 induced an increase in cleaved caspase 9 in the acute myeloid cell lines, having no clear effect on T-cell derived Jurkat cells. This increase is time dependent, starting at 4h after treatment and showing a significant increase of caspase 9 activity around 6h post-treatment.

We also studied the effect of ZINC69391 on caspase 8 activation using caspase 8 specific antibodies. As expected, its activation was also time-dependent showing a significant increase in caspase 8 cleavage at later time points compared to caspase 9 activity (Figure 7B). These results are consistent with a caspase 8 cleavage as a consequence of advanced stages of the apoptotic program initiated by mitochondrial damage instead of an activation of death receptor pathway [33, 34].

### ZINC69391 displays no proapoptotic effect on human lymphocytic and monocytic normal cells

**Figure 8:**
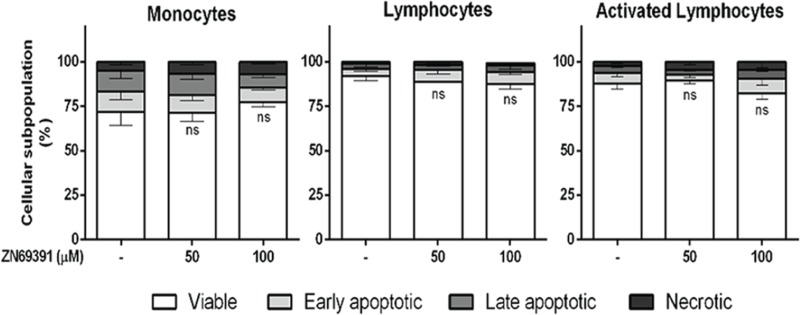
Compound selectivity assessed in normal peripheral blood mononuclear cells (PBMC) Phosphatidylserine exposure was measured by annexin V binding assay in monocytes, unstimulated lymphocytes or phytohemagglutinin A activated (i.e. proliferating) lymphocytes after 24h treatment with ZINC69391 at the indicated concentrations or to 0.05% (v/v) DMSO vehicle control group. Data represent the mean ± SD (n > 3). ns. No significant differences (ns).

We next sought to determine whether this proapoptotic effect of ZINC69391 was selective for cancer cells. Normal peripheral blood mononuclear cells (PBMC) from healthy donors were stained using annexinV/PI. As shown in Figure 8, after 24h treatment with ZINC69391 50 μM or 100 μM, there were no significant changes in cell viability compared to vehicle-treated cells. In the same way, Phytohemagglutinin A activated (i.e. proliferating) lymphocytes were also not sensitive to ZINC69391 action.

Interestingly, the concentration of ZINC69391 that induced the highest apoptotic effect in leukemic cells failed to induce cytotoxicity on PBMC from healthy donors.

### ZINC69391-derived 1A-116 analog displayed increased potency in leukemic cell lines and showed a profound pro-apoptotic effect on patient-derived leukemic cells

**Figure 9:**
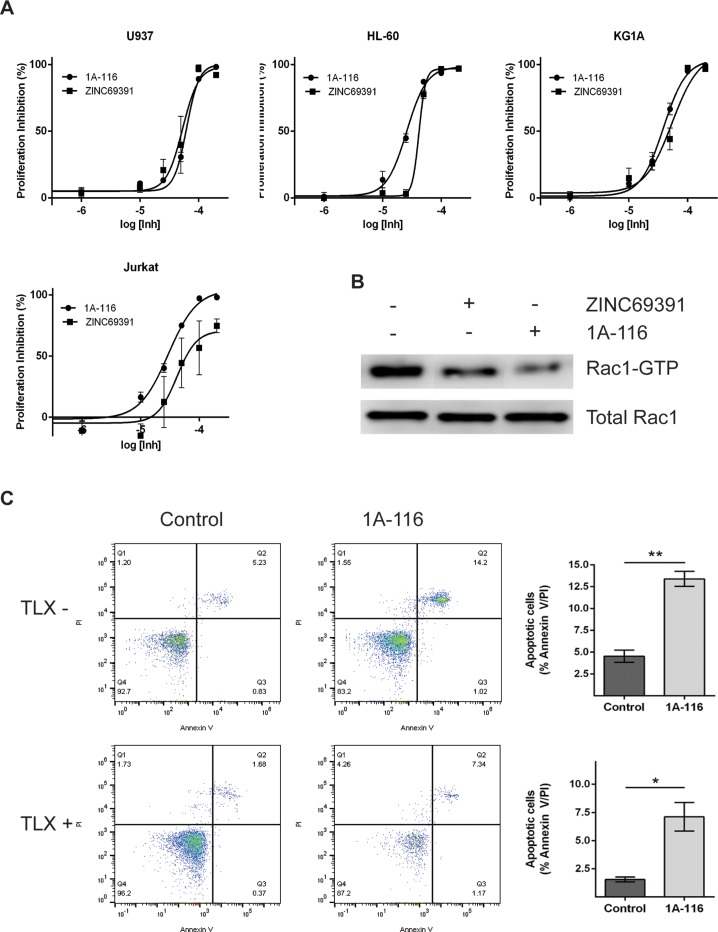
1A-116 is a more potent Rac1 inhibitor and shows proapoptotic activity on patient-derived leukemia cells **(A)** Cells were cultured in the presence of increasing concentrations (100 nM to 300 μM) of ZINC69391 or 1A-116 for 48h. Cell viability was determined by MTS assay and reported as a percent of proliferation inhibition respect to vehicle treated cells (n>3). **(B)** HL-60 cells were treated with 50 μM ZINC69391 and 50 μM 1A-116 for 24h and cell lysates were subjected to a pull down assay. Both drugs reduced Rac1-GTP levels, being 1A-116 more potent than the parental drug (n=3 independent experiments). **(C)** Patient-derived leukemia cells were treated with 50 μM 1A-116 for 24h. Apoptotic cell population was analyzed using annexinV staining by flow cytometry. ^*^p<0.05; ^**^p<0.01.

We have previously described the development of 1A-116, a novel analog of ZINC69391, which showed to be more potent than the parental compound in glioma and aggressive breast cancer models [18, 19]. To evaluate the effect of 1A-116 on cell proliferation in leukemic cells, we measured cell viability by the MTS metabolic assay. 1A-116 inhibited cell proliferation in a concentration-dependent manner, showing enhanced efficacy in Jurkat cells reaching a 100% inhibition in cell proliferation. Moreover, in the more undifferentiated cellular models of myeloid leukemia (HL-60 and KG1A), 1A-116 showed a left shift in the concentration response curves indicating an increased potency in its antiproliferative activity (Figure 9A).

Additionally, we compared the effect of both compounds on Rac1 activation levels. As seen in Figure 9B, ZINC69391 and 1A-116 treatment on HL-60 cells decreased Rac1-GTP levels. Interestingly, 1A116 showed a more potent effect on Rac1 activation compared to ZINC69391 parental drug. This effect is in line with previous our reports in other cancer models.

Finally, we evaluated the effect of 1A116 compound on recently established patient-derived leukemic cell cultures. For this purpose, we used two independent primary cultures of leukemic cells from lymphocytic lineage. One of these cell cultures is TLX+, while the other is TLX-. TLX is a an oncogene and its expression defines a distinct subgroup of T cell acute lymphoblastic leukemia, being TLX1+ tumors prone to aneuploidy and showing marked defects in the activation of normal mitotic checkpoints [35]. We evaluated the effect of 50 μM 1A-116 treatment in both cell cultures. Interestingly, 1A-116 treatment induced apoptosis in both cell types, being TLX- cells more sensitive. This effect is shown in Figure 9C, where TLX- cells had a significant increase in the apoptotic population after 24h treatment, while a similar effect is shown for TLX+ cells after 48h treatment. It is important to mention that TLX+ cells treated for 24h showed no apoptotic effect (data not shown).

## DISCUSSION

Rac1 has long been recognized as an important regulatory protein in different physiological and disease processes [36]. We have previously shown the effect of Rac1 inhibition by ZINC69391 and 1A-116 in breast cancer and glioma models, but Rac1 represents attractive innovative target for many other types of cancers [37]. The present work shows the effect of ZINC69391 and 1A-116 compound in hematopoietic malignancies. The rationale that led us to explore this cancer indication was based in evidence showing that Rac1 is over-expressed in leukemia patients [15, 36]. Moreover, CD34+ cells isolated from patients with AML showed high levels of activated Rac1 and this signaling pathway has been identified as a critical mediator of stem cell and stroma interaction [10, 12]. Additionally, loss of Rac1 impairs engraftment, homing, localization and proliferation of hematopoietic stem cells/progenitor cells [38].

Ours results show that ZINC69391 is able to reduce the activation of Rac1 GTPase in HL-60 cells. This effect correlates with the programmed cell death induction observed as consequence of growth cell inhibition on a panel of human leukemic cells. These results are in line with previous reports that use dominant negative constructs, siRNA strategies as well as other Rac1 inhibitors to suppress Rac1 activity [12, 36, 39].

Remarkably, ZINC69391 showed low micromolar IC50 values in all cell lines tested, independently on their hematopoietic cell differentiation stage or lineage. In fact, previous reports showed that leukemic cells with MLL (Mixed Lineage Leukemia) rearrangements were specifically vulnerable to Rac1 inhibition, while cell lines such as HL-60, U937 and Jurkat were more resistant [10, 40]. Our data show that ZINC69391 treatment inhibited cell proliferation of leukemic cells despite the fact that all cell lines tested here do not harbor MLL rearrangements. These results show a differential *in vitro* potency compared to other Rac1 inhibitors and this could be a key issue for *in vivo* efficacy in further animal studies. It could be interesting to determine the effect of this new family of Rac1 inhibitors in leukemic cells bearing MLL rearrangements. We would expect higher anticancer potency against this type of tumors.

We next studied the underlying mechanisms of the antiproliferative effect induced by ZINC69391. In previous work on solid tumors, we identified that ZINC69391 antiproliferative effect was mainly due to cell cycle arrest in G0/G1 and apoptosis induction [19]. Interestingly, cell cycle analysis in leukemic cells lines showed a G2/M arrest, which correlated with the antiproliferative activity exerted by the compound. It has been described that Rac1 inactivation is associated with altered cell cycle progression by impaired centrosomal activation in G2 phase [20, 21]. Additionally, the appearance of a sub-G0 population of cells in these DNA fluorescence histograms suggests the detection of apoptotic cells on the basis of their reduced DNA content. In this regard, ZINC69391 induced apoptosis in all the cell lines evaluated. The stimulation of apoptosis exerted by ZINC69391 was caspase-dependent, since pro-caspase 3 cleavage was observed in a concentration dependent manner. Interestingly, although Jurkat cells did not show changes in annexin V staining after 50μM ZINC69391 treatment, they did show an increase in cleaved caspase 3 at high concentration (100 μM). These results show that lymphoid Jurkat cells are more resistant to ZINC69391-induced apoptosis than AML cells.

We found that ZINC69391 induces the activation caspase 9, triggering the intrinsic pathway of apoptosis. Only after caspase 9 stimulation, caspase 8 activation is evidenced. Additionally, the treatment of leukemia cells with ZINC69391 resulted in mitochondrial membrane function loss and an increase in Bcl-2 phosphorylation in a time dependent manner, both events associated to apoptosis induction.

One possible mechanism contributing to the pro-apoptotic effect of Rac1 inhibition in leukemic cells may be through the Rac1-dependent regulation of NF-κB transcriptional activity. Rac1 can mediate the activation of several oncogenic pathways, including NF-κB activation [41, 42]. NF-κB can, in turn, induce the transcription of various proteins, being a central regulator of lymphocyte proliferation, survival and development [43]. We observed that ZINC69391 provoked a significant reduction of mRNA levels of IL-8 on HL-60 cells, suggesting that IL-8 expression may be Rac1-dependent in HL-60 cells; while this effect was not shown by Jurkat cells. These results are in line with the observation that Jurkat cells are more resistant to ZINC69391 treatment, and it may be due to a compensation mechanism associated to Jurkat´s PTEN null phenotype. The tumor suppressor gene PTEN (phosphatase and tensin homolog) is an important negative regulator of PI3K-Akt pathway. PTEN null cells maintain PI3K signaling constitutively activated, increasing cancer susceptibility, tumor cell proliferation and drug resistance in multiple hematological malignancies [44, 45]. Since NF-κB transcriptional activity is controlled by parallel pathways, including PI3K/Akt, IL-8 increased levels in Jurkat cells could be related to this characteristic phenotype.

Evasion of apoptosis is primarily driven by upregulation from pro-survival members of the Bcl-2 family, especially Bcl-2, Bcl-xL and Myeloid cell leukemia 1 (Mcl-1) [46]. Indeed, we show that pharmacological inhibition of the Rac1 signaling pathway by ZINC69391 in HL-60 cells inhibited Mcl-1 expression, showing no effect on Bcl-2 and Bcl-xL expression. Interestingly, the same treatment on Jurkat cells shows a significant increase in the expression of these three pro-survival proteins. This upregulation may be a part of a resistance mechanism, where Jurkat cells overexpress the pro-survival proteins to overcome, without success, Rac1 inhibition. In this sense, several reports indicate that Bcl-2 and Bcl-xL [47, 48] enhanced expression is mediated through a NF-κB-dependent mechanism, while Mcl-1 is a downstream target of PI3K signaling pathway [49]. Interestingly, despite the fact that we observed diverse cell responses and sensitivities to Rac1 inhibition in different hematopoietic cell lineages, in all cell lines tested we ultimately observed increased apoptosis after ZINC69391 treatment.

Although Rac1 has proven to be an interesting molecular target in AML and other myeloproliferative diseases [10, 50], Rac1 also plays an important role in normal hematopoietic cell homeostasis [12, 51]. In this sense, ZINC69391 treatment did not promote cell death program on normal peripheral blood mononuclear cells, including Phytohemagglutinin A activated (i.e. proliferating) lymphocytes. Moreover, we show that 1A-116 compound significantly induces apoptosis in two patient-derived leukemic cells. Thus, we can speculate that Rac1 signaling in leukemia cancer cells mainly contributes to the acquisition of the transformed phenotype, while normal mononuclear cells do not rely entirely on this pathway and are able to compensate Rac1 inhibition. These results are also in line with a previous report, where Rac1 protein levels in leukemia patients were significantly higher than that in normal donors and this overexpression contributes to cell proliferation and leukemia progression [15]. In agreement, it has been previously suggested that transformed cells become addicted to Rac1 signaling for survival and leukemia maintenance [16].

It is important to note, that in this work most experiments were performed using 50 μM and 100 μM ZINC69391. Although this compound was used to study the mechanism of action and the effect on cancer cells of a novel Rac1 inhibitor family, we are aware of its limitations for further clinical development. For this reason, we tested the effect of the ZINC69391-derived analog 1A-116. This analog shares high structural homology with ZINC69391 compound and both have shown to exert their biological effect by the same mechanism of action in different cancer models. In line with previous published data in solid tumors models, 1A-116 showed increased potency in AML cell, showing an enhanced reduction of Rac1-GTP levels compared to the parental drug. This molecular effect is reflected in an augmented antiproliferative activity as well.

In agreement with the relevant role of Rac1 signaling pathway in several malignancies, the inhibitory effects displayed for ZINC69391 and 1A-116 highlight their potential as new therapeutic agents. In this sense it would be very interesting to evaluate these inhibitors in Kaposi´s sarcoma, where Rac1 seems to play an important role in tumorigenesis and it is overexpressed in AIDS-associated Kaposi's Sarcoma lesions [52]. Our evidence suggests that the use of Rac1 inhibitors could be a useful tool for novel therapeutic management of these tumors.

Finally, Rac1 signal transduction is an interesting molecular target in acute leukemia and its inhibition by pharmacological agents such as ZINC69391 and 1A-116 induces the cell death program. This effect was exclusively observed in cancer cell lines and patient-derived cells when compared to normal PBMC. Future *in vivo* studies using pharmacological agents targeting Rac1 pathway in combination with other therapeutic agents could point out a new treatment option for leukemic malignancies.

## MATERIALS AND METHODS

### Reagents and antibodies

ZINC69391 (Figure 1A) was purchased from Enamine database and solubilized in dimethyl sulfoxide (DMSO). 1A-116 (Figure 1B) was synthesized as reported and solubilized in aqueous vehicle. Stock solutions were stored at -20°C until use and final concentration for each experiment comprised less than 0.1%. Cell culture medium RPMI-1640 and antibiotics were obtained from Sigma Chemical Company (St. Louis, MO) and FBS from Natocor (Argentina). Anti-Bcl-2 and anti-αTubulin antibodies were purchased from Santa Cruz Biotechnology (Santa Cruz, CA, USA); anti-caspase 8 was from Cell Signaling Technologies (Massachusetts, USA) and anti-caspase 3 antibody from Neuromics (Edina, MN, USA). Anti-CD5 Monoclonal Antibody (UCHT2), PE-Cyanine7, was obtained from eBioscience. A horseradish peroxidase-conjugated goat anti-mouse and anti-rabbit were used as the secondary antibody (Vector and Santa Cruz Biotechnology, respectively). Annexin V-FITC/PI apoptosis detection kit was obtained from BD Biosciences Pharmingen (San Diego, CA, USA) and caspase 9 substrate was obtained from AnaSpec (Fremont, CA, USA).

### Cell culture and synchronization

Human leukemia cell lines U937, HL-60, KG1A and Jurkat were obtained from American Type Culture Collection (ATCC) and grown in RPMI-1640 medium (Sigma Aldrich Co.) supplemented with 10% fetal bovine serum (FBS) and 50 μg/ml Gentamicin in a humidified 5% CO_2_ atmosphere at 37°C.

Peripheral blood mononuclear cells (PBMC) were obtained from heparinized samples of healthy donors isolated by centrifugation on Ficoll-Hypaque. Cells were cultured at 37°C in a humidified atmosphere with 5% CO_2_ in RPMI-1640 medium, supplemented with 10% FBS and 50 μg/ml Gentamicin. For activation of the peripheral T cells, 2.0×10^6^ cells/ml were incubated with Phytohemagglutinin A at a concentration of 1.0 μg/ml for 48h before treatment with ZINC69391. Blood samples from normal volunteers were obtained after written informed consent in accordance with the Declaration of Helsinki. These studies were approved by the institutional review board of the National Academy of Medicine of Buenos Aires.

Primary T-ALL cells of the TLX1+ and TLX– subtypes were kindly provided by Dr. Xosé Bustelo. Cells were processed for genetic and flow cytometry characterization and rapidly stored in liquid nitrogen. For expansion, cells were thawed, cultured in the presence of feeder layers of OP9-DL1 cells in MEM α containing IL-7 (5 ng/mL, Peprotech), Flt3L (5 ng/mL, Peprotech) and 20% FBS for 48h, and injected into sublethally-irradiated (2 Gy) 6- to 8-week-old NOD-Scid IL2rg^null^ mice (NSG, Jackson Laboratory). Engrafted T-ALL blasts (CD5+CD7+CD45+) were collected by preparative flow cytometry from the thymi, spleens, and bone marrows of the recipient mice 10 weeks later. Cells were then frozen in liquid nitrogen and, when needed, cultured on OP9-DL1 cells as indicated above.

The TLX1+ T-ALL cells were TCRα/β–, TCRγ/δ–, CD45++, CD34–, TdT+ (58%), icCD3+, mCD3low, CD7++, CD5++, CD2+ (78%), CD4+, CD8–, CD1a+ (90%), CD10+, CD13–, CD33–, CD56–, CD123– and CD117–.

The TLX– T-ALL cells were mTCRα/β+ (100%), CD4+ (31%), CD8–, CD5+, CD7+, CD45+, IL7R–, ICN1–, and TLX–. In addition to the cytogenetic analyses, the TLX status of primary tumor cells was confirmed by qRT-PCR both before and after expansion in immunocompromised mice.

For cell synchronization at G0/G1, cells were serum-starved for 7h at 37°C and thereafter relieved into cell cycle by addition of 10% FBS. Before seeding, viability of cell lines and PBMC were tested by Trypan Blue assay. Cells were used only if viability was higher than 90%.

### MTS assay

Cell proliferation was determined by a colorimetric assay using CellTiter 96 AQueous Non-Radioactive Cell Proliferation Assay (Promega, USA) according to the manufacturer's instructions. For MTS assay, cells growing in exponential phase were seeded at 2.0×10^4^ cells/well in a 96-well plate and incubated in an atmosphere of 5% CO_2_ at 37°C. Cells were exposed to serial dilutions of ZINC69391, 1A-116 or 0.1% (v/v) DMSO (vehicle control group). After incubation for 48h, 20 μl of MTS was added to each well and further incubated for 2h at 37°C. The absorbance was measured at 490 nm using the FlexStation 3 microplate reader (Molecular Devices Inc., USA).

Half maximal inhibitory concentration 50 (IC50) values were calculated with GraphPad Prism software (GraphPad Software Inc., USA) using the sigmoidal dose-response function and expressed as IC50 (μM). Assays were carried out in triplicate and at least three independent experiments were conducted.

### Analysis of cell cycle phases distribution by flow cytometry

Synchronized cell populations were treated with 50 μM of ZINC69391 or 0.05% (v/v) DMSO (vehicle) for 15h. After treatment, cells were harvested, washed with ice-cold PBS, fixed overnight by addition of 70% (v/v) ethanol and stored at -20°C for a minimum of 24h. On the day of flow cytometry analysis, cell suspensions were washed with ice-cold PBS and re-suspended in 50 μl RNase A (100 μg/ml) at room temperature for 15 min.

Propidium Iodide was added to a final concentration of 20 μg/ml and incubated in dark at room temperature for 20 min. Cell cycle phase distributions were analyzed by FACS Scan Flow Cytometer (Beckton-Dickinson CA, USA). Data from at least three independent experiments were analyzed using ModFit software (VeritySoftware House Inc., Topsham, ME, USA) to determine the fractions of cells in the subG0/G1, G0/G1, S and G2/M phases from cell cycle distribution.

### Determination of apoptosis markers

#### Annexin V/PI binding assay

Starved cell populations were plated in 12-well plates at a density of 5.0×10^5^ cells/ml and cultured with 50 μM of ZINC69391 or vehicle (0.05% DMSO) in complete medium for 24h. After washing with ice-cold PBS, 2.0×10^5^ cells were incubated with FITC-labeled annexin V and PI according to the manufacturer's instructions (BD Biosciences Pharmingen, San Diego, CA, USA) and analyzed by a FACS Scan Flow Cytometer (Becton-Dickinson CA, USA).

Patient-derived T-ALL cells were treated with 50 μM 1A-116 for 24h, stained using the annexin V–fluorescein propidium isothiocyanate detection kit (Immunostep), and apoptosis determined in the population of CD5 positive cells using flow cytometry.

Annexin V is a calcium-dependent phospholipid-binding protein that has an affinity for phosphatidylserine (PS) which in early-stage apoptosis becomes translocated to the extracellular membrane leaflet. In late stage apoptosis, the cell membrane loses integrity thereby allowing annexin V to also access PS in the interior of the cell. In these cases the addition of a viability dye such as propidium iodide (PI) can be used to resolve these late-stage apoptotic and necrotic cells [53].

#### Caspase 3 activity assay

Cells growing in exponential phase were seeded in 6-well plates and treated with 50 and 100 μM ZINC69391 or vehicle (DMSO) in complete medium during 12h and 24h. Cells were then harvested and processed according to CASP3C caspase 3 colorimetric assay kit (Sigma Chemical Co. St. Louis, MO, USA). Absorbance at 405 nm, due to hydrolysis of the peptide substrate acetyl-Asp-Glu-Val-Asp p-nitroanilide (Ac-DEVD-pNA), was measured using the FlexStation 3 microplate reader (Molecular Devices Inc., USA) and caspase 3 activity was expressed as OD405 value.

### RT-PCR and quantitative real-time PCR

Total RNA was isolated from HL-60 and Jurkat cells using Quick-Zol reagent (Kalium Technologies) following the manufacturer's instructions. For the first-strand cDNA synthesis, 1 μg of total RNA was reverse-transcribed using the High Capacity cDNA Reverse Transcription kit (AB) with random primers. Quantitative real-time PCR (qPCR) was performed in triplicate on the Rotor Gene Q cycler (Qiagen) using the resulting cDNA, the HOT FIREPol EvaGreen qPCR Mix Plus (Solis Biodyne) for product detection, and the following primers: Human Bcl-2 (B-cell lymphoma 2; NM_000633.2) forward, 5′-GGATGCCTTTGTGGAACTGTAC-3′ and reverse, 5′- TTCACTTGTGGCCCAGATAGG-3′; Bcl-xL (B-cell lymphoma extra large; NM_138578.2) forward, 5′-GGTCGCATTGTGGCCTTTTT-3′ and reverse, 5′- GCTCTAGGTGGTCATTCAGGT-3′; Mcl-1 (Myeloid cell leukemia 1; NM_021960.4) forward, 5′- GCTTCGGAAACTGGACATCAA-3′ and reverse, 5′- CCAGTTTGTTACGCCGTCG-3′; IL-8 (Interleukin 8; NM_000584) forward 5′-5´-CTGCGCCAACACAGAAATTA-3´-3′ and reverse 5´-ATTGCATCTGGCAACCCTAC-3´; and human β-Actin (βAct) forward, 5′-GGACTTCGAGCAAGAGATGG-3′ and reverse 5′-AGCACTGTGTTGGCGTACAG-3′. The cDNA was amplified by 45 cycles of denaturing (10 s at 95°C), annealing (10 s at 60°C), and extension (10 s at 72°C) steps. The specificity of each primer set was monitored by analyzing the dissociation curve, and the relative GILZ, THBD or SLC19A2 mRNA quantification was performed using the comparative ΔΔCt method using β-Actin as the housekeeping gene.

### Mitochondrial membrane permeability evaluation (MMP)

In order to assess mitochondrial membrane potential in U937 cells, 6.0×10^5^ cells/ml were seeded in 48-well plates and treated with 50 μM of ZINC69391 at different times. After treatment, cells were harvested, centrifuged and incubated in the dark with 10 nM of the probe DiOC6 in RPMI-1640 for 20 min. Fluorescence was analyzed by flow cytometry (ʎ ex/ʎem = 488/530nm) using FACS Scan Flow Cytometer (Becton-Dickinson CA, USA) and results were analyzed with ModFit software (Verity). According to the results obtained for U937 cell line, HL-60, KG1A and Jurkat were also evaluated for 3, 5 and 7h with 50 μM of ZINC69391.

### Preparation of cell lysates and Western blot analysis

Cells were washed in PBS and lysed in 50 mM Tris–HCl pH 6.8, 2% SDS, 100 mM 2-mercaptoethanol, 10% glycerol and 0.05% bromophenol blue and sonicated to shear DNA. Cellular proteins from total cell lysates (20 μg) were electrophoresed on 8–15% SDS polyacrylamide gel and transferred to nitrocellulose membranes. Blots were blocked with 5% non-fat powdered milk in TBS containing 0.05% Tween-20 and probed with the indicated primary antibodies followed by horseradish-peroxidase-conjugated secondary antibodies. Reactivity was developed by enhanced chemiluminescence (ECL) according the manufacturer's instructions (Amersham Life Science, England).
